# Unorthodox superficial palmar arch observed in a South Indian cadaver: a case report

**DOI:** 10.4076/1757-1626-2-6362

**Published:** 2009-07-16

**Authors:** Venkata Ramana Vollala, Somayaji Nagabhooshana, Mohandas Rao, Bhagath Kumar Potu, Narendra Pamidi, Srinivasa Rao Bolla

**Affiliations:** 1 Department of Anatomy, Melaka Manipal Medical CollegeManipalIndia; 2 Department of Anatomy, Asian Institute of Medicine, Science and TechnologySungai Petani, KedahMalaysia; 3 Department of Anatomy, Kasturba Medical CollegeManipalIndia; 4 Department of Anatomy, Mamata Medical CollegeKhammamIndia

## Abstract

Variations in formation of the superficial palmar arch are common. A classic superficial palmar arch is defined as direct continuity between the superficial branch of the ulnar artery and superficial palmar branch of the radial artery. During routine dissection classes to undergraduate medical students we have observed formation of superficial palmar arch solely by superficial branch of ulnar artery without any contribution from the radial artery or median artery. Knowledge of the anatomical variations of the arterial pattern of the hand is crucial for safe and successful hand surgery.

## Introduction

Superficial palmar arch (SPA) is an arterial arcade which lies beneath the palmar aponeurosis and in front of the long flexor tendons, lumbrical muscles and palmar digital branches of the median nerve. SPA is the major blood supply to the hand. Additional circulation for the palm may come from the median artery or interosseous arterial system. The radial and ulnar arteries form four circuits in the hand, the anterior and posterior carpal arches at the level of the carpal bones and the superficial and deep palmar arches at the mid palmar level [1]. The anastomoses between radial and ulnar arteries in the palm play a significant role in diseases of the palm through collateral circulation. Various anomalous patterns in the superficial arch of the hand have been reported. Among these variations is the superficial palmar branch of the radial artery passing deep to the flexor retinaculum to form the SPA [2], absence of the SPA [3] and incomplete development of the SPA [4]. Knowledge of the frequency of anatomical variations of the arterial pattern of the hand is crucial for safe and successful hand surgery [5].

## Case presentation

**Figure 1.**
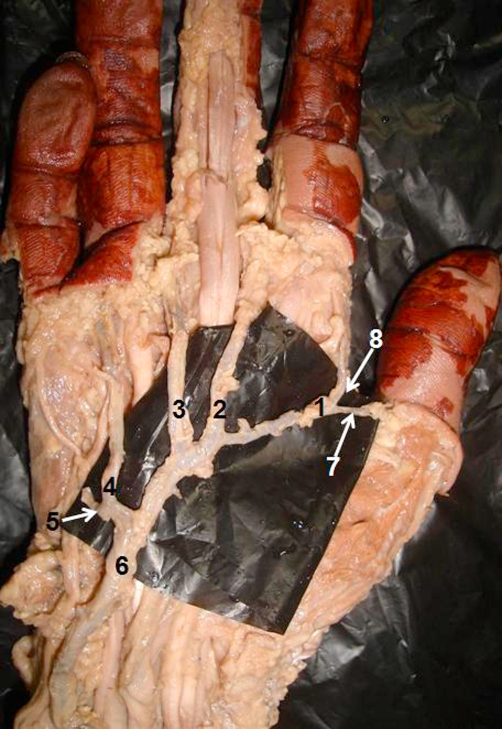
Dissection of the right palm showing the superficial palmar arch formed solely by superficial branch of ulnar artery: **(1-4)** common palmar digital arteries; **(5)** branch to supply ulnar side of the little finger; **(6)** superficial branch of ulnar artery; **(7)** princeps pollicis artery; **(8)** radialis indicis artery.

During routine dissection of the right upper limb of a 45-year-old South Indian male cadaver, we observed SPA formed exclusively by superficial branch of the ulnar artery (Figure 1). The superficial palmar branch of the radial artery terminated in the thenar muscles without any contribution to the SPA. The superficial branch of the ulnar artery gave origin to four common palmar digital arteries to supply the digits. The first common palmar digital artery divided into radialis indicis and princeps pollicis arteries to supply the radial side of the index finger and both sides of thumb, there were no branches from the deep palmar arch to supply the index finger and thumb. The second, third and fourth common palmar digital arteries divided into digital branches to supply the sides of second, third and fourth web spaces and the fourth common palmar digital artery in addition gave a branch to supply ulnar side of the little finger.

## Discussion

Superficial branch of ulnar artery solely forming SPA is reported [5-7]. McCormack et al. [8] in their comprehensive study on the arterial pattern of 750 hands did not find the origin of the princeps pollicis and radialis indicis arteries from the SPA. Erbil et al. [9] described five cases in which the SPA provided the blood supply to the thumb and index fingers through the princeps pollicis and radialis indicis arteries. None of the above studies showed that princeps pollicis and radialis indicis arteries can arise from superficial branch of ulnar artery. According to Ikeda et al. [7] the artery arising from the SPA to supply the first web space can be called as the first common palmar digital artery. So, in the current study it can be said that the superficial branch of ulnar artery forming SPA gave origin to four common palmar digital arteries. The first common palmar digital artery originating from superficial branch of ulnar artery is a rare anomaly.

The use of radial arteries as an arterial bypass conduit is an invasive procedure which is becoming popular among various medical centers. The greatest risk associated with harvesting the radial artery is ischemia of the soft tissues of the hand. But in patients where ulnar artery is the main blood supply to the first web space the least number of complications may be expected.

The SPA is the center of attraction for most of the surgical procedures and traumatic events in the hand. The hand surgeon should keep in mind this kind of variations before performing surgical procedures such as, arterial repairs, vascular graft applications, and free and/or pedicled flaps. SPA is an anastomosis fed mainly by the ulnar artery. When ulnar artery is occluded, the viability of the structures in palm supplied by the ulnar artery depends on the efficacy of the collateral circulation. In our finding there was no anastomosis between the ulnar artery and radial or median or interosseous arteries. So ulnar artery occlusion in cases like ours there will be no collateral flow of blood to meet the metabolic demands of the palmar tissue i.e., results in acute ischaemia, manifested by rest pain and / or gangrene.

During surgical procedures of thumb in the cases similar to ours ligation of radial artery may not be sufficient to stop the profuse bleeding since major blood supply was coming from the superficial palmar arch. Several techniques are used to identify and locate any unusual vessel in the upper limb, including Doppler ultrasound, the modified Allen test, pulse oximetry and arterial angiography.

## Abbreviation

SPA: Superficial palmar arch
